# Effects of sphingosine-1-phosphate receptor modulators on remyelination in multiple sclerosis: evidence from *in vitro* experiments, animal models and human studies

**DOI:** 10.3389/fimmu.2026.1895118

**Published:** 2026-09-09

**Authors:** Vito A.G. Ricigliano

**Affiliations:** 1 Neurology Unit, GHNE Paris-Saclay Hospital, Orsay, France; 2 Université Paris-Saclay, UNIACT, Neurospin - INSERM UMR 1129, CEA, Gif-sur-Yvette, France

**Keywords:** multiple sclerosis, oligodendrocyte progenitor cell (OPC), oligodendrocytes, remyelination, S1P receptor modulators

## Abstract

Sphingosine-1-phosphate (S1P) receptor modulators are a class of therapeutic agents used for the treatment of multiple sclerosis (MS). There are four S1P modulators currently approved by the Food and Drug Administration/European Medical Agency (FDA/EMA): fingolimod, siponimod, ponesimod, and ozanimod. Beyond their well-established immunomodulatory properties, accumulating evidence suggests that these molecules exert direct effects on oligodendrocyte progenitor cells (OPCs) and mature oligodendrocytes, promoting myelin repair through multiple mechanisms. *In vitro* studies show enhanced OPC differentiation and process extension, while *in vivo* models demonstrate increased remyelination, improved myelin protein expression, and functional recovery. Imaging studies in people with MS suggest increased myelin repair over time. Potential mechanisms underlying the observed effects involve selective modulation of S1P receptor subtypes (particularly S1PR1, S1PR3, and S1PR5). This comprehensive review summarizes available preclinical and clinical evidence on the role of S1P modulators in remyelination in the context of MS and shows that fingolimod, siponimod and ponesimod possess emerging promise in this field.

## 1 Introduction

Sphingosine-1-phosphate (S1P) is a bioactive lipid mediator that regulates diverse cellular processes through five G-protein-coupled receptors (S1PR1-5). These receptors exhibit distinct tissue distribution patterns and are associated to different G-protein subtypes, resulting in varied downstream intracellular signalling cascades. S1PR1, S1PR2, and S1PR3 are widely expressed, with S1PR1 being the most extensively characterized and involved in lymphocyte trafficking, while S1PR4 and S1PR5 show more restricted distribution. S1PR5 is particularly enriched in oligodendrocytes and natural killer cells (1). Upon ligand binding, S1P receptors activate multiple intracellular pathways, including mitogen-activated protein kinases (MAPK), phosphoinositide 3-kinase (PI3K)/Akt, and Rho guanosine triphosphate-phosphohydrolase (GTPases), which collectively regulate cell survival, proliferation, migration, and differentiation (2). In the central nervous system (CNS), S1P signalling plays critical roles beyond lymphocyte migration. Neural cells, including oligodendrocytes, astrocytes, and neurons, are characterized by distinct S1P receptor profiles that enable cell-type-specific responses to S1P gradients (1, 3). This differential expression pattern of receptors suggests that S1P modulators may exert variable effects within the CNS, with relevant implications for normal physiology and disease.

On a therapeutic viewpoint, a class of compounds modulating S1P receptors has been developed in the field of multiple sclerosis (MS) and represented a major advance in the treatment landscape, with fingolimod being the first oral immune-active MS therapy approved by the Food and Drug Administration (FDA), dating back to September 2010. These agents act as functional antagonists of the target receptors, by inducing S1PR internalization and degradation, particularly affecting S1P1 on lymphocytes, thereby preventing lymphocyte egress from lymphoid organs and reducing CNS infiltration. Four S1P modulators are currently used for MS treatment: fingolimod (Gilenya^®^), siponimod (Mayzent^®^), ozanimod (Zeposia^®^), and ponesimod (Ponvory^®^). Fingolimod (FTY720) was the pioneer of this class of molecules, commercialised worldwide as disease-modifying treatment (DMT) for relapsing MS (RMS). Following phosphorylation to its active form (FTY720-P), it acts as a non-selective S1P receptor modulator with effects on S1PR1, S1PR3, S1PR4, and S1PR5 subtypes (4). Its broad receptor profile contributes to both immunomodulatory and potential CNS-directed effects, but this comes with cardiac adverse events related to S1PR1 receptor activation on atrial myocytes, mimicking vagal stimulation. Siponimod (BAF312) is a second-generation, selective S1PR1 and S1PR5 modulator approved in some countries for active secondary progressive MS (SPMS) (5). Its profile was designed to maintain immunomodulatory efficacy while potentially enhancing CNS effects through engagement on oligodendrocytes (6). Ozanimod is a selective S1PR1 and S1PR5 modulator with a receptor profile similar to Siponimod, approved in some countries to treat RMS. Ponesimod is a selective S1PR1 modulator with functional antagonist properties on this receptor subtype and is the most recently licensed of his class for RMS treatment. Its high selectivity for S1PR1 distinguishes it from earlier-generation compounds and may influence its therapeutic properties on oligodendrocytes (7).

**Figure 1:**
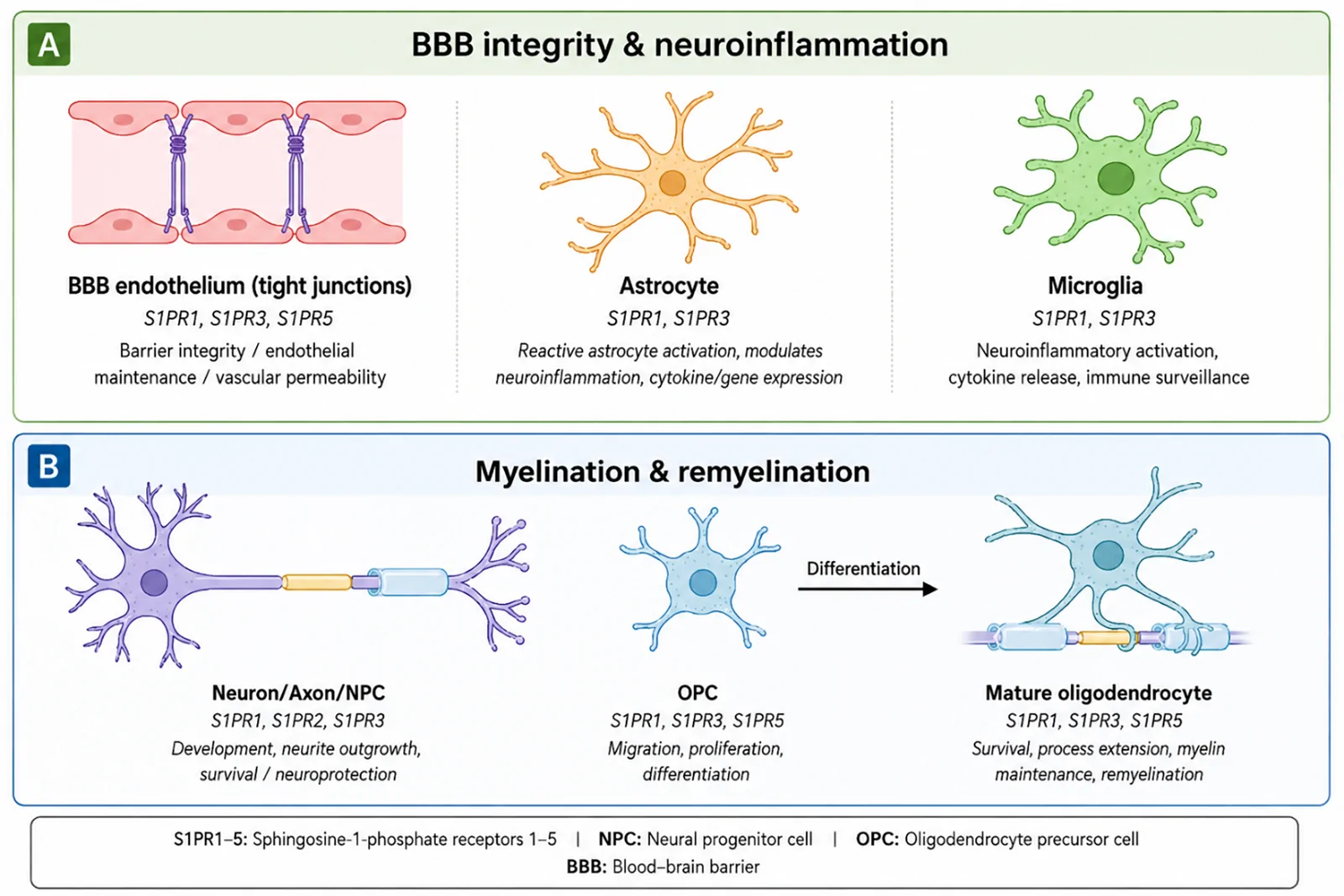
Distribution and functions of sphingosine-1-phosphate receptors (S1PRs) across central nervous system cell populations involved in neuroinflammation and myelination.

The effects of S1PR modulators on the egress of lymphocytes from lymph nodes is undoubtedly the most well know mechanisms of action of this category of MS medications. However, as already mentioned, oligodendrocyte lineage cells express multiple S1P receptor subtypes, with S1PR1, S1PR3, and S1PR5 being particularly relevant for their biology (8). Oligodendrocyte progenitor cells (OPCs) represent the primary source of remyelinating oligodendrocytes in the adult CNS (9). The differentiation of OPCs into mature, myelinating oligodendrocytes is a tightly regulated process involving proliferation, migration to demyelinated lesions, and terminal differentiation accompanied by myelin sheath wrapping around uncovered axons (10). Demonstration of S1PR modulators’ brain penetration, although at different proportions varying from one molecule to another (11), encourages the exploration of their potential direct effects on CNS cells such as OPCs, oligodendrocytes, astrocytes, microglia (1, 11–13) (**Figure 1**). Indeed, data suggests that S1PR modulators, other than reducing neuroinflammation, may preserve blood–brain barrier (BBB) integrity, and protect neurons and oligodendrocytes (14, 15).

More in detail (**Figure 1A**, BBB integrity and neuroinflammation), within the neurovascular unit, S1PR1 is the predominant receptor expressed by brain endothelial cells, where its activation stabilizes adherens and tight junctions, helps barrier function, and limits leukocyte transmigration into the CNS (16, 17). S1PR3, and under specific conditions S1PR5, further modulate endothelial barrier and vascular responses (12, 16). In astrocytes, S1PR1 and S1PR3 regulate reactive astrogliosis, NF-κB-dependent inflammatory gene expression, and cytokine/chemokine secretion (12, 18), whereas in microglia these receptors influence migration, activation, phagocytosis, and the production of inflammatory mediators that shape innate immune responses during neuroinflammation (16, 17). Specifically considering the processes of myelination and remyelination – namely, focusing on neuronal substrates and myelin sheath-producing cells (**Figure 1B**, myelination and remyelination) – S1PR expression is dynamically regulated throughout oligodendrocyte lineage progression, coordinating distinct stages of myelination and remyelination. In neurons and neural progenitor cells (NPCs), S1PR1, S1PR2, and S1PR3 influence neuronal survival, neurite extension, synaptic plasticity, and differentiation through PI3K/Akt, ERK/MAPK, and Rho GTPase signalling pathways (16, 17, 19). Early OPCs predominantly express S1PR1, which promotes proliferation, migration, and survival, while S1PR3 contributes to cytoskeletal remodelling and chemotactic migration (19, 20). As differentiation proceeds, S1PR1 expression progressively declines, whereas S1PR5 expression markedly increases, becoming the predominant receptor in mature oligodendrocytes (19, 20). Activation of S1PR5 enhances oligodendrocyte survival, process extension, and myelin sheath stability through Rac1- and RhoA-dependent cytoskeletal signalling, thereby promoting myelin maintenance and remyelination following demyelinating injury (19, 20). This developmental transition from S1PR1- to S1PR5-dominant signalling provides a mechanistic basis for the direct neuroprotective and pro-remyelinating effects of CNS-penetrant S1PR modulators.

Remyelination following the demyelinating insult of MS remains a major unmet need in this disease, since there are currently no approved therapies to promote myelin repair and preserve electric axonal function. Given the expression of some S1PR on oligodendroglial lineage cells, compounds acting on them could support myelin preservation and, possibly, remyelination in neurodegenerative and demyelinating disorders (15–21). This review summarizes the current knowledge on the potential effects of all four S1PR modulators on remyelination in experimental models and in people with MS (PwMS), analysing findings from *in vitro* experiments, animal models of disease and human - mainly imaging - studies in patients.

## 2 Methods

The literature review presented here was conducted through PubMed search until May 2026, using the following combination of search terms: ‘fingolimod’, or ‘siponimod’, or ‘ozanimod’, or ‘ponesimod’ and ‘myelin’, or ‘remyelination’, or ‘oligodendrocytes’, or ‘oligodendrocyte progenitor cells’, or ‘OPCs’. The extracted list of articles was carefully reviewed for validation. The selected literature was then enriched with human magnetic resonance imaging (MRI) findings on remyelination under S1PR modulators through the screening of relevant communications (posters/oral presentations) at international MS conferences (ECTRIMS, ACTRIMS).

## 3 Results

**Table 1** summarizes the available evidence regarding the remyelinating potential of S1PR modulators across *in vitro*/ex vivo systems, animal models and human studies.

**Table 1:** Remyelination evidence for sphingosine-1-phosphate receptor modulators across preclinical and clinical settings.

Drug	*In vitro* studies	Animal models	Human studies
Fingolimod	• ↑ O1/MBP+ and O4/GC+ cells in human foetal OPC cultures; promotes axonal ensheathment (2) • In human brain organoids, increases MBP levels and reduces inflammatory markers (20). • Pro-differentiating effects persist after S1PR desensitisation, suggesting sustained intracellular signalling (14)	**Organotypic cerebellar slices** • ↑ MBP staining ~2-fold at 14 days; ↓ phagocytic microglia (15) • ↑ Remyelinated axons at 0.3 mg/kg (dose- dependent therapeutic window) (16) • High local spinal cord concentration → ↑ lesion size; cytotoxic at ≥10% (16) **EAE** • ↑ OPC proliferation and differentiation (17) • + NSC co-treatment: ↑ myelination, prolonged survival in shiverer mice (effects independent of immune context) (18) **Cuprizone** • No remyelination or neuroprotection when given after demyelination (19)	• ↓ VEP latency vs IFN-β at 6 months (optic neuritis trial) (21) • q-Space myelin map imaging: signs of remyelination in optimal responders (22) • MTR: ↑ lesional MTR from month 6; ↑ NAWM and GM MTR at month 24 (vs natalizumab stabilisation) (23)
Siponimod	• LPC cerebellar slices: ↓ demyelination 97.4% → 53.8%; ↓ IL-6 ~50% via S1PR5 (24) • ↑ Mature oligodendrocyte survival and process extension; no ↑ OPC proliferation (pro-survival/differentiation profile) (24)	**Xenopus laevis** • Among most efficient remyelinating compounds; bell-shaped dose-response (25, 26) **Cuprizone** • ↑ Remyelinated axons 2.19-fold; ↑ pro- regenerative microglia; ↑ oligodendroglial differentiation markers (26) • ↑ MBP and MAG expression; ↑ OLIG2+ density; promotes OPC differentiation over proliferation (27) • + Vitamin D3: ↑ myelin recovery; ↓ M1 microglia in corpus callosum (28) **Cuprizone + Rapamycin** • 25–40% ↓ myelin protein loss (demyelinating phase); ~25% ↑ myelinated axons by EM (remyelinating phase) (6) **EAE-Optic Neuritis (EAEON)** • ↓ Clinical scores, retinal degeneration, optic nerve demyelination; remyelinating effect confirmed with therapeutic (post-onset) dosing (26)	• EXPAND post-hoc (n=1037 vs 523): ↑ MTR in lesions, NAWM, GM, and normal-appearing brain tissue at 12 and 24 months vs placebo (29)
Ponesimod	• ↑ OPC differentiation (O4+) with ponesimod alone; further ↑ with S1PR5 agonist (A971432) co-treatment (7)	**Cuprizone** • ↓ VEP latency (functional remyelination of optic nerve); reversal of working memory deficits (7) • ↑ MBP by IHC and ↑ myelinated axons with improved sheath morphology by EM in corpus callosum (7) • Both preventive and therapeutic administration reduce demyelination and show remyelination with prolonged exposure (30)	OPTIMUM post-hoc (sT1w/T2w ratio): • Preservation of myelination in cingulum and remaining NAWM; trend toward remyelination in NAWM at 2 years (31) • Less NAWM modifications compared with teriflunomide • Lesional myelin changes not assessed (31)
Ozanimod	*No data available*	**EAE** • ↓ Inflammation, demyelination, and neurodegeneration (32) **Cuprizone** • ↓ Axonal degradation and myelin loss (preventive setting) (32) • No stimulation of remyelination post-intoxication (32)	*No data available*

EAE, experimental autoimmune encephalomyelitis; EM, electron microscopy; GC, galactocerebroside; GM, grey matter; IHC, immunohistochemistry; LPC, lysolecithin; MAG, myelin-associated glycoprotein; MBP, myelin basic protein; MTR, magnetization transfer ratio; NAWM, normal-appearing white matter; NSC, neural stem cell; O1/O4, oligodendrocyte markers 1 and 4; OPC, oligodendrocyte progenitor cell; S1PR, sphingosine-1-phosphate receptor; sT1w/T2w, standardized T1-/T2-weighted ratio; VEP, visual evoked potential.

↑ indicates increase, ↓ indicates decrease. Experimental settings are indicated in bold.

### 3.1 Fingolimod (FTY720)

Being the first S1P modulator developed, fingolimod is the most extensively studied one with respect to remyelination.

#### 3.1.1 *In vitro* studies on human OPCs

Most studies investigating the direct effects of fingolimod on oligodendrocyte lineage cells have been conducted using rodent OPCs, mixed glial cultures, or organotypic slice cultures, with relatively few studies using isolated human OPCs.

Using primary human foetal OPCs, it was shown that the active metabolite FTY720-P directly modulates OPC morphology and survival through S1P receptor signalling. Mechanistically, the authors showed that early exposure to FTY720-P induced S1P5/RhoA-dependent process retraction, whereas prolonged exposure promoted S1P1-mediated process extension and enhanced cell survival through activation of the PI3K/Akt and ERK1/2 pathways (8). These opposing responses were associated with cyclic regulation of S1P receptor expression, suggesting that temporal changes in receptor signalling govern the transition from initial cytoskeletal remodelling to subsequent process extension and survival.

In a complementary study (22), fingolimod elicited stage- and dose-dependent effects within the oligodendrocyte lineage. Compared with mature oligodendrocytes, OPCs exhibited greater morphological plasticity and a more robust pro-survival response, indicating that developmental changes in S1P receptor expression influence cellular responsiveness to the molecule. Regarding its direct effects on oligodendrocyte differentiation, daily FTY720-P application increased oligodendrocyte marker 1 (O1)/myelin basic protein (MBP) double-positive cells and promoted axonal ensheathment, while also increasing oligodendrocyte marker 4 (O4)/galactocerebroside (GC) double-positive cells in dissociated OPC cultures (2). Interestingly, the differentiation-promoting effects persisted even under conditions of external S1P receptor desensitization. This paradoxical finding suggests that FTY720 induces persistent intracellular signalling even after SPR internalization, directly promoting OPC differentiation through sustained activation of downstream pathways (23).

#### 3.1.2 Animal model studies

##### 3.1.2.1 Organotypic cerebellar slice cultures

Experiments on organotypic cerebellar slice cultures were performed to investigate fingolimod’s effects on remyelination in a system that maintains physiological cell-cell interactions while minimizing systemic immune influences. Following lysolecithin-induced demyelination, fingolimod treatment significantly enhanced remyelination, increasing MBP staining by 1.97 to 2.00-fold over control when giving it for 14 days post-lysolecitine. While OPC numbers showed a trend toward increase (9 ± 1 cells/field with fingolimod versus 5 ± 0 in controls), fingolimod treatment additionally decreased significantly phagocytic microglial cells from 13 ± 0 to 5 ± 2 at 5 days and from 4 ± 1 to 1 ± 1 at 14 days.

The positive effect of this reduction in inflammatory microglia on remyelination may rely on the generation of a more permissive environment for myelin repair (26). The ability of fingolimod to enhance remyelination following focal lysolecithin-induced demyelination in mice was subsequently confirmed by other studies. Fingolimod treatment, particularly at lower doses (0.3 mg/kg), increased the number of remyelinated axons new myelinating cells compared to vehicle-treated controls. This dose-dependent effect suggests an optimal therapeutic window of repetitive treatment with FTY720 for remyelination promotion (24), with beneficial repair effects behind its anti-inflammatory properties.

Nonetheless, despite these encouraging results, in a rat model where FTY720 was delivered locally via injection into spinal cord lesions, results unexpectedly showed a two-fold increase in the size of demyelinated lesions, suggesting that high local concentrations may eventually be cytotoxic to oligodendrocytes. Indeed, while lower doses of about 0.1%–1% stimulated cell growth, high ones (10%) significantly inhibited proliferation, possibly by activating different pathways.

##### 3.1.2.2 Experimental autoimmune encephalomyelitis, shiverer mice and cuprizone-induced toxic demyelination

Multiple studies have examined fingolimod’s effects in the experimental autoimmune encephalomyelitis (EAE) inflammatory model of MS. Zhang et al. demonstrated that fingolimod treatment promotes proliferation and differentiation of OPCs in mice with EAE (25). In a subsequent study, Zhang et al. investigated combination therapy with fingolimod and transplanted neural stem cells (NSCs) (27). Using three myelination models with little to no inflammatory microenvironment (organotypic slice culture, postnatal oligodendrocyte maturation, shiverer mouse model), they found that fingolimod drives NSC differentiation into oligodendrocytes and promotes myelination (27). In shiverer mice, which lack functional MBP and serve as a model of abnormal myelination, FTY720 and NSC co-treatment prolonged survival and improved sensorimotor function, showing greater myelination than NSCs alone. Importantly, these effects were observed in a non-immune context like that of shiverer mice, seem to suggest that fingolimod’s promyelinating effects act independently of its immunomodulatory properties (27). However, later Nystad et al. reported that fingolimod does not promote remyelination or neuroprotection in the cuprizone model when administered after demyelination (28). This negative finding suggests that its efficacy may depend on the specific demyelinating insult, timing of administration of the toxic product or of development of abnormal myelin, or presence of residual inflammatory components. In that sense, the post-natal metabolic oligodendrocyte injury without significant lymphocytic infiltration of the cuprizone model may explain the differential response compared to inflammatory models and shiverer mice.

#### 3.1.3 Human brain organoid models and findings in people with MS

Recent advances in three-dimensional culture systems have enabled investigation of fingolimod effects in human brain organoids that incorporate astrocytes, microglia, and oligodendrocytes. These neuroinflammatory human brain organoids provide physiologically relevant platforms for drug screening that better recapitulate human CNS architecture and cellular interactions. Studies using these systems have confirmed effects on oligodendrocyte lineage cells (29). In a recent work, for instance, it was shown that fingolimod and all its derivatives decreased inflammatory markers and favoured neuroprotection, with a 2.9-fold increase in the levels of MBP in favour of remyelination (29). Human studies have shown a potential beneficial effect of this molecule on optic nerve remyelination versus IFN-β treatment, with a statistically larger reduction of visual evoked potential latency at 6 months compared to baseline in the fingolimod group (30). A pilot analysis assessing remyelination through q-space myelin map imaging in few people with MS (PwMS) treated with fingolimod indicated that optimal responders showed some signs of remyelination and no newly demyelinated lesions, thus suggesting enhanced myelin repair activity (31). However, solid conclusions cannot be drawn give the very small size of the analyzed group. Another study assessing myelin content through magnetization transfer ratio (MTR) over 2 years in PwMS treated with fingolimod (n=25) or natalizumab (n=30) found that the S1PR modulator gradually increased lesional MTR at subsequent timepoints starting from month 6 compared to baseline, while natalizumab stabilized it. Moreover, at later timepoints (month 24), fingolimod also induced an MTR increase in the normal-appearing white matter (NAWM) and the grey matter (GM) These findings suggest an enhancement of focal and diffuse tissue repair, with different timing, in PwMS treated with this medication (32).

### 3.2 Siponimod (BAF312)

Siponimod is a second-generation, selective S1PR1 and S1PR5 modulator that has demonstrated remyelinating properties in multiple preclinical models. Its selectivity profile was specifically designed to maintain immunomodulatory efficacy through S1PR1 engagement while potentially enhancing CNS effects through S1PR1 and S1PR5 activity on oligodendrocytes. Studies demonstrate that siponimod promotes remyelination by preserving myelin, supporting oligodendrocyte differentiation, and fostering a regenerative microglial environment, with reproducible benefits across ex vivo and mechanistic models (33).

#### 3.2.1 *In vitro* and ex vivo studies

In ex vivo LPC-demyelinated cerebellar slice cultures, the medication significantly reduced demyelination from 97.4% in untreated slices to 53.8% (34). This effect on myelin preservation was associated with reduced interleukin-6 release by ~50%, indicating direct protection of oligodendrocytes through S1PR5 signalling rather than immune modulation, and with activation of ERK1/2 and Akt signalling pathways in astrocytes. Siponimod fostered mature oligodendrocyte survival, with no significant increase in OPC proliferation, suggesting a pro-survival/pro-differentiation effect rather than a mitogenic one.

Using lysolecithin-demyelinated foetal rat CNS aggregate cultures, it was further demonstrated that siponimod significantly enhanced remyelination, as evidenced by increased MBP expression, whereas selective S1P1 agonism alone did not improve myelin repair, indicating that activation of S1P5 is likely required for the drug’s promyelinating effects (35).

#### 3.2.2 Animal models

##### 3.2.2.1 Xenopus laevis demyelination model

Screening for promyelinating compounds in a Xenopus transgenic line allowing conditional ablation of oligodendrocytes showed that siponimod was one of the most efficient molecules promoting remyelination, acting through S1PR5 (36). A conditional demyelination model in Xenopus laevis tadpoles to assess dose-response relationships demonstrated that siponimod improved remyelination compared to controls, following a bell-shaped dose-response curve meaning high concentrations were less efficient than low concentrations (33).

##### 3.2.2.2 Cuprizone model

Multiple studies have investigated siponimod’s effects in the cuprizone model of toxic demyelination. Dietrich et al. demonstrated increased remyelination in cuprizone-treated mice receiving siponimod (33). The study employed multiple complementary approaches including mouse MRI to quantify remyelination, providing objective imaging-based evidence of myelin repair. Siponimod treatment increased remyelinated axons by 2.19-fold and induced a significant shift of microglia toward a pro-regenerative phenotype, accompanied by higher expression of oligodendroglial differentiation markers, supporting indirect enhancement of remyelination. Krüger et al. conducted detailed histological analyses of siponimod-treated mice following seven weeks of cuprizone intoxication. Siponimod treatment significantly increased MBP and myelin-associated glycoprotein (MAG) expression compared to vehicle-treated mice. Densities of OLIG2+ oligodendrocytes significantly recovered in siponimod-treated mice, while vehicle-treated mice showed no such recovery. Moreover, these results showed that siponimod may promote OPC differentiation rather than proliferation (37). The findings in the cuprizone model are particularly significant given that this model produces metabolic oligodendrocyte injury without primary lymphocytic infiltration. The observation that siponimod enhances remyelination in this environment suggests that direct CNS effects may be independent of peripheral immunomodulation.

Al-Otaibi et al, investigated combination therapy with vitamin D3 and siponimod in the cuprizone model. This study examined not only remyelination but also microglial activation patterns, providing insights into the cellular mechanisms underlying siponimod’s regenerative effects (38). Findings showed that siponimod promoted myelin recovery and attenuated the M1 microglia phenotype in the corpus callosum at early and late remyelination stages.

##### 3.2.2.3 Cuprizone + rapamycin model

Tiwari-Woodruff et al. employed a toxic demyelination model combining cuprizone diet with rapamycin to assess siponimod’s effects during both demyelinating and remyelinating periods (6). In demyelinating groups, siponimod-treated mice exhibited a significant 25% to 40% decrease in the loss of myelin proteins, mature oligodendrocytes, and axon damage. During the remyelinating phase, electron microscopy analysis revealed nearly 25% increased numbers of myelinated axons in siponimod-treated groups, although this effect was not detected by standard immunohistochemistry. This discrepancy highlights the importance of using multiple complementary techniques to assess remyelination.

##### 3.2.2.4 EAE and EAE-Optic neuritis

In EAE mice, continuous intracerebroventricular administration of siponimod (0.45 μg/day for 4 weeks) ameliorated EAE by exerting direct CNS effects independent of peripheral lymphocyte sequestration (39). Treatment preserved parvalbumin-positive GABAergic interneurons, restored inhibitory synaptic transmission in the striatum, and reduced microgliosis and astrogliosis. Siponimod also suppressed the release of pro-inflammatory mediators, including IL-6 and RANTES, from activated microglia, thereby limiting neuroinflammation through central mechanisms rather than via peripheral immunomodulation (39). The EAEON model enabled assessment of siponimod’s effects on both structural and functional outcomes. Siponimod attenuated clinical scores, reduced retinal degeneration, and improved visual function after both prophylactic and therapeutic treatment. Histological analyses revealed that inflammatory infiltrates and demyelination of the optic nerve were reduced. Remarkably, demyelination was reduced not just with prophylactic administration, but also when therapeutic treatment was initiated following disease onset, demonstrating genuine remyelinating rather than purely protective effects (33).

#### 3.2.3 Imaging studies in PwMS

*Post-hoc* analysis of the EXPAND trial in people with secondary progressive MS assessed the effect of siponimod (n=1037) versus placebo (n=523) in normalized MTR change, reflecting myelin dynamics, from baseline to month 12/24 in lesions, NAWM, GM and normal-appearing brain tissue (40). Authors found that siponimod was associated with myelination and structural integrity across all the explored regions over the study period.

### 3.3 Ponesimod

Ponesimod is a highly selective S1PR1 functional antagonist (41). While clinical experience with ponesimod is more limited compared to fingolimod and siponimod, emerging preclinical evidence suggests remyelinating potential (7).

#### 3.3.1 *In vitro* studies

To investigate direct effects on oligodendrocyte lineage cells, cultured primary mouse OPCs were treated with ponesimod alone or in combination with A971432, an S1P5 mono-selective modulator. Both ponesimod alone and the combination treatment significantly increased OPC differentiation, as assessed by O4 immunocytochemistry (marker of late-stage OPCs and immature oligodendrocytes, indicating progression toward a myelinating phenotype), without impairing precursor cell mobility and recruitment to sites of injury. However, the observation that combining ponesimod with an S1P5 agonist further enhanced differentiation suggests that functional antagonism of S1PR1 may remove an inhibitory signal, while S1PR5 activation might provide a positive differentiation stimulus (7).

#### 3.3.2 Cuprizone model

In cuprizone mice model, preventively, ponesimod was administered simultaneously with cuprizone, while therapeutically, it was given after cuprizone exposition. Results showed reduced demyelination and signs of remyelination with prolonged exposure (13). Willems et al. (7) conducted a comprehensive investigation of ponesimod’s effects in the cuprizone mouse model of demyelination. This study employed both *in vivo* and *in vitro* approaches to characterize ponesimod’s remyelinating properties. *In vivo*, ponesimod treatment decreased the VEP latency, indicating functional remyelination and restoration of axonal conduction velocity in the optic nerve. Additionally, ponesimod reversed cuprizone-induced working memory deficits, demonstrating cognitive benefits, possibly associated brain tissue preservation and myelin repair. Histological analyses using MBP immunohistochemistry and transmission electron microscopy confirmed increased myelination in the corpus callosum of ponesimod-treated mice. Electron microscopy, the gold standard for assessing myelin ultrastructure, revealed increased numbers of myelinated axons and improved myelin sheath morphology (7).

#### 3.3.3 Imaging studies in PwMS

Evidence for ponesimod is currently limited. A *post-hoc* analysis of the OPTIMUM Phase III trial of ponesimod versus teriflunomide valuated myelination changes over two years using standardized T1-weighted/T2-weighted (sT1w/T2w) ratio, a metric sensitive to microstructure, whose changes are predominantly driven by myelin content in WM regions. Analysis of sT1w/T2w in the corpus callosum, cingulum, and remaining NAWM showed that teriflunomide was associated with significant myelination decrease across all examined regions at both time points, whereas ponesimod showed preservation of myelination in the cingulum and remaining NAWM, with only modest demyelination in the corpus callosum. Moreover, ponesimod treatment was associated with trends toward remyelination in the remaining NAWM at 24 months (42). To note, lesional myelin content modifications over time were not assessed in the study.

### 3.4 Ozanimod

Ozanimod is a selective S1PR1 and S1PR5 modulator with a receptor profile similar to siponimod (12, 43). The lack of published studies specifically investigating ozanimod’s remyelinating properties represents a significant knowledge gap. Given its structural and pharmacological similarity to siponimod, including selectivity for both S1PR1 and S1P5 (12), it is plausible that ozanimod may possess remyelinating properties.

#### 3.4.1 Animal models

In EAE, exposure to ozanimod reduced inflammation, demyelination and neurodegeneration. In the cuprizone mice, ozanimod prevented axonal degradation and myelin loss but did not enhance remyelination after intoxication and toxin withdrawal, likely due to the exclusive S1P1 receptor engagement by free drug in this model, while the remyelinating effect is mediated by S1P5 receptor (44). Indeed, the authors highlighted that ozanimod has limited activity at mouse S1P5 compared with human S1P5, suggesting that rodent studies may underestimate potential human S1P5-mediated effects on oligodendrocyte biology.

#### 3.4.2 *In vivo* studies in PwMS

There are currently no published imaging and/or electrophysiology studies in PwMS exploring whether a remyelinating effect of ozanimod exists *in vivo* in humans.

## 4 Discussion and future directions

Fostering remyelination pathways *in vivo* remains a major challenge of MS care: despite multiple efforts and several clinical trials, there are currently no molecules approved with the indication of promoting myelin repair in PwMS. In this scenario, it is of great interest to explore the remyelination potential of known DMT with a broader spectrum of action, going beyond immune modulation, such as the S1P modulators. The four drugs of this class approved for the treatment of MS differ substantially in their receptor selectivity profiles, which likely influence their remyelinating properties. Fingolimod is a non-selective modulator with activity at S1PR1, S1PR3, S1PR4, and S1PR5. Its broad receptor profile contributes to robust remyelinating effects mediated primarily through S1PR1, S1PR3 and S1PR5. The extensive evidence for fingolimod, spanning human OPC cultures, multiple animal models, and functional outcomes, establishes it as the benchmark S1P modulator for remyelination. Siponimod is selective for S1PR1 and S1PR5. This selectivity profile was specifically designed to maintain immunomodulatory efficacy through S1PR1 while enhancing CNS effects through S1PR5 and S1PR1 engagement on oligodendrocytes. The consistent demonstration of remyelinating effects across multiple models, including the non-inflammatory cuprizone model, confirms that S1PR1/5 selectivity is sufficient for promoting myelin repair. The bell-shaped dose-response relationship observed with siponimod suggests that optimal S1 PR engagement requires careful dose titration. Ponesimod is highly selective for S1PR1. Its remyelinating effects appear to result from functional antagonism of S1PR1, removing an inhibitory signal for OPC differentiation. Ozanimod is selective for S1PR1 and S1PR5, similar to siponimod. However, the absence of published remyelination studies prevents assessment of its actual efficacy on myelin repair.

While the overall evidence for S1P modulator remyelinating effects is substantial, several conflicting findings warrant discussion: Nystad et al. reported that fingolimod does not promote remyelination or neuroprotection in the cuprizone model when administered after demyelination. This contrasts with positive findings for siponimod in the same model. Moreover, the efficacy of S1P modulators appears to vary across different demyelinating models. Effects are most consistent in inflammatory models (EAE, EAEON) and organotypic cultures, with more variable results in toxic demyelination models (cuprizone, lysolecithin). Additional studies *in vitro* and in animal models are needed to better investigate the remyelination effect of the two most recent S1PR modulators, ponesimod and ozanimod. More recently, several additional S1PR modulators, such as ceralifimod and amiselimod, have been developed. However, direct effects of either compound on myelin repair, OPCs, or oligodendrocytes have not yet been demonstrated (1).

To note, most of the current evidence on remyelination promotion comes from preclinical models or *in vitro* human cell systems. Whether these findings translate to meaningful remyelination and persistent beneficial effects over time in PwMS remains uncertain. Clinical studies specifically designed to assess remyelination outcomes in S1PR modulators-treated PwMS would provide valuable translational evidence. These could benefit of electrophysiology measurements, such as the assessment of VEP latency changes over time in people treated with S1PR modulators versus an active comparator, such as teriflunomide, or of imaging sequences sensitive to myelin (e.g., myelin water fraction, MTR, χ-separation, radial diffusivity) to specifically measure dynamic remyelination in the treated group, with additional clinical outcomes to demonstrate whether the observed modifications, other than statistically significant, are also clinically relevant and maintained over time. The integration of multiple techniques to explore remyelination (e.g. MRI, VEP, cerebrospinal fluid biomarkers) would provide answers even more reliable in this context (45).

Some precisions must be provided regarding the methodology used in the review, which generated subsequent limitations in the findings presented here. Indirect effects of S1PR on myelin recover, such as those induced by reduction of astrogliosis or modulation of the inflammatory microenvironment, were voluntarily not extensively discussed, for the sake of space. Moreover, when presenting imaging findings in PwMS, we specifically selected studies performed using MRI proxies of myelin content, not including those measuring the effects of S1PR modulators on brain volume preservation or atrophy. Indeed, while being a marker of neuroprotection – therefore, with possible contribution of myelin restoration – this metric has a broader meaning, being also sustained by additional mechanisms such as neuro-axonal preservation, reduced inflammation etc, going beyond the scope of this review. Lastly, indirect effects of S1PR modulators on myelination acting through the periphery, e.g. via the gut (where these molecules also play a role) (46) and affecting the gut-brain axis – which has been shown to influence OPCs in mice through bacteria-produced molecules like butyrate, indole lactate or acetate (47) – were not discussed since they remain speculative, but grant further investigation.

In conclusion, evidence coming from translational settings supports a role for S1PR modulators in promoting remyelination in MS, but additional, larger studies, specifically designed to explore this effect *in vivo* in PwMS, are needed to confirm the clinical value of these findings.
